# A Nitroreductase‐Activatable Lapachol Against *Bacillus subtilis* Unveils Antimicrobial Specificity

**DOI:** 10.1002/cmdc.202500997

**Published:** 2026-03-13

**Authors:** Ivonne R. Lopez‐Miranda, Tianyi Ma, Joshua N. Milstein, Andrew A. Beharry

**Affiliations:** ^1^ Department of Chemical & Physical Sciences University of Toronto Mississauga Mississauga Canada; ^2^ Department of Chemistry University of Toronto Toronto Canada; ^3^ Department of Physics University of Toronto Toronto Ontario Canada

**Keywords:** antimicrobial resistance, lapachol, nitroreductase, prodrug

## Abstract

Lapachol is a natural product with antimicrobial activity. Though promising for combating antimicrobial resistance, lapachol has toxicity to mammalian cells, which will cause severe side effects in vivo. To mitigate this, we developed alapachol prodrug which is dependent on the action of nitroreductase—an enzyme expressed in bacteria but not healthy mammalian cells. We observed release of lapachol in vitro by purified nitroreductase and in the gram‐positive bacterial strain, *Bacillus subtilis*. Colony formation assays in *Bacillus subtilis* indicated release of lapachol, achieving comparable levels of toxicity as free lapachol. Lastly, minimal toxicity of our prodrug was observed in mammalian cell culture, demonstrating a >10‐fold selectivity for bacteria over healthy mammalian cells.

## Graphical abstract

A nitroreductase‐activatable lapachol prodrug overcomes toxicity to mammalian cells b selectively releases the antimicrobial natural product only in bacteria, addressing antimicrobial resistance. Enzymatic reduction in *Bacillus subtilis* triggers self‐immolative uncaging to restore antibacterial activity while sparing healthy cells.

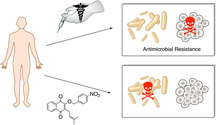

## 1 Introduction

Antibiotics were discovered over 100 years ago and they are credited with having drastically changed modern medicine, extending the average human lifespan by 23 years [1]. However, misuse of these compounds has led to the rapid spread of antimicrobial resistance (AMR), adversely affecting the effectiveness of clinically established treatments [2]. Specifically, AMR accounts for 9% of all global deaths and has emerged as a crucial global health issue, resulting in treatment failures, longer hospitalizations, and sky‐rocketing healthcare costs [3]. Tackling AMR is a global public health emergency requiring drug alternatives.

A solution to AMR is the development of stronger antibiotics. However, many of these have been linked to various adverse outcomes, stemming from the induction of mitochondrial dysfunction [4] and DNA lesions [5] in mammalian cells, leading to downstream effects and toxicity to the host. Patients being treated for bacterial infections may, consequently, suffer severe side effects from these drugs.

Just as many of the earliest antibiotics were derived from natural sources (e.g. penicillin, streptomycin, quinolines), modern‐day treatments look to established natural products as a safer and efficient source to treat microbial infections. Lapachol is a naphthoquinone that was first discovered in 1882 and has since demonstrated a wide spectrum of therapeutic activities, amongst which are anticancer, anti‐inflammatory, antimalarial, antiseptic, antitumor, antiviral, bactericidal, fungicidal, insectifugal, and pesticidal [6]. In terms of its anti‐bacterial behavior, lapachol interferes with topoisomerases, which are critical for DNA replication in the cell [6], and with the electron transport system, inhibiting the cell's respiratory mechanism [7].

Previously, the antimicrobial efficacy of another naphthoquinone, β‐lapachone, was investigated in the form of a photoactivatable prodrug that successfully demonstrated release of the natural product upon light irradiation and displayed targeted effects in cancer cells and biofilms [8, 9]. However, the light used to uncage the protecting group in these cases was in the UV‐range, which has short penetration depths and is cytotoxic for surrounding healthy tissue.

Nitroreductases (NTRs) are a well‐studied class of enzymes known to reduce nitrogroups to amine moieties with the use of nicotinamide adenine dinucleotide (NADH) as a cofactor, most recently used in prodrug applications to activate the release of therapeutics [10, 11]. NTRs are found in bacteria (e.g. *Escherichia coli*, *Staphylococcus aureus*, and *Bacillus subtilis*) but not in healthy mammalian cells [10, 12]. Therefore, by appending a self‐immolative nitrogroup to the drug of choice, NTR‐mediated reduction would lead to the release of the drug only when bacteria harboring NTR are present [11].

Recently, a nitroreductase‐activatable photosensitizer was synthesized for selective antimicrobial photodynamic therapy [13]. A drawback to this design is the need for light which has poor penetration depths to initiate the cytotoxic behavior from the photosensitizer and illicit antimicrobial activity. Taking inspiration from these previous prodrugs, we sought to synthesize a compound that will display selective antimicrobial activity with safe dosages without toxic effects on mammalian cells or the need for light.

Lapachol has demonstrated activity towards a wide variety of microbial strains. Particularly, lapachol targets gram‐positive bacteria effectively. Recently, bacterial cell studies were conducted in the NTR‐expressing model gram‐positive strain, *Bacillus subtilis*, where lapachol exhibited a minimal microbicidal concentration comparable to the reference antibiotic, gentamycin [14, 15]. We hypothesize that if an NTR trigger group can be added to lapachol to mask its activity, then selective targeting of infectious NTR‐containing bacteria over mammalian cells would occur, eliminating unnecessary mitochondrial and DNA damage to mammalian cells. With the broad number of bacterial strains that express NTR, and the rising health concerns caused by AMR bacteria, the development of novel treatments can further build upon the foundation for efficient antimicrobial therapies.

## 2 Results and Discussion

**FIGURE 1:**
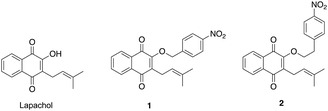
Lapachol compared to the NTR prodrug, **1**, and control compound, **2**.

Lapachol was synthesized from the commercially available 2‐hydroxy‐1,4‐naphthoquinone using a previously reported synthetic route [16]. Lapachol contains a free hydroxyl, which has been found to be crucial for protein interactions. Given literature precedent that lapachol is reduced by cytochromes [17], we reasoned that adding a nitrobenzyl moiety to the free hydroxyl can readily perturb lapachol–enzyme interaction [18]. Nucleophilic substitution of lapachol with the corresponding 4‐nitrobenzyl bromide in basic conditions yielded prodrug **1** and a control compound **2** (Figure 1).

**FIGURE 2:**
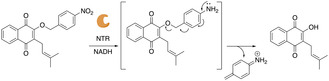
Mechanism of action for **1**. Upon incubation with NTR, the nitro group is reduced to an amine, leading to the spontaneous 1,6‐elimination releasing free lapachol.

Compound **1** contains a nitro group which will readily be reduced to an amine upon incubation with NTR and its cofactor, NADH. Production of the amine is expected to trigger spontaneous 1,6‐elimination leading to release of free lapachol (Figure 2).

**FIGURE 3:**
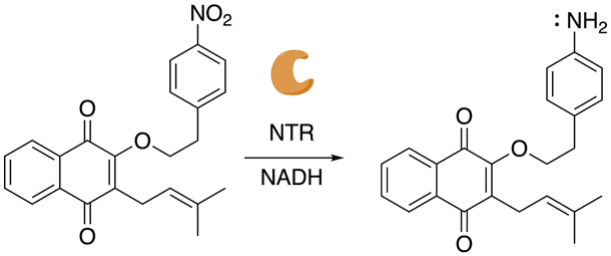
Mechanism of action for **2**. Upon reaction with NTR, the nitrogroup is reduced to an amine, but due to the presence of an ethylene linker, no 1,6‐elimination and release of lapachol is expected.

Compound **2** was synthesized with a nitrophenylethyl group, which still contains a reducible nitro group but due to the additional methylene compared to **1**, is expected to not undergo 1,6‐elimination and release lapachol (Figure 3). Therefore, **2** will confirm that any bacterial cell death observed with **1** is due to NTR‐dependent release of lapachol.

**FIGURE 4:**
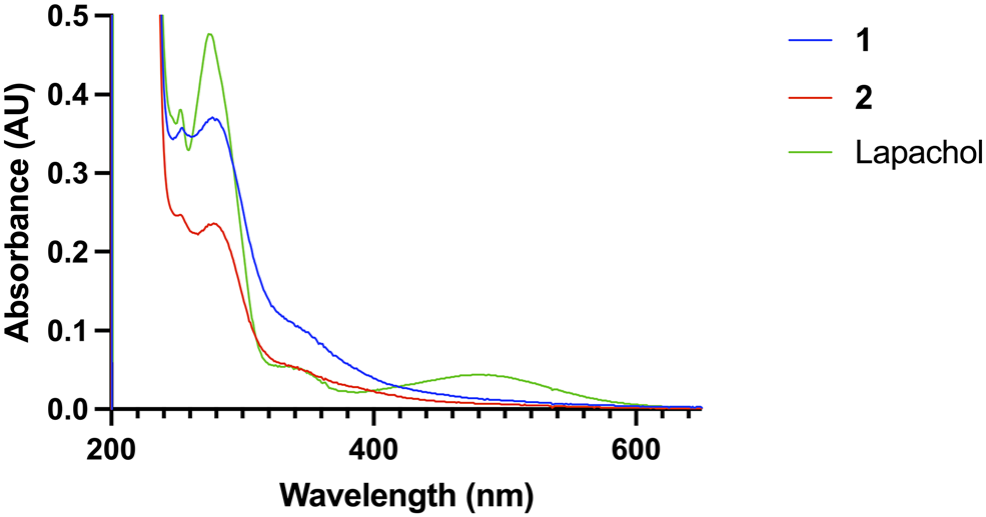
UV–Vis absorbance spectra of 10 µM of lapachol, **1**, and **2** in PBS. Lapachol displays a characteristic absorbance band at 480 nm (*λ*_max_).

The spectral properties of the compounds were compared to lapachol in PBS (Figure 4). Lapachol has a characteristic absorbance band at 480 nm, which is no longer present upon attachment of the 4‐nitrophenyl group as in **1** and **2.**

**FIGURE 5:**
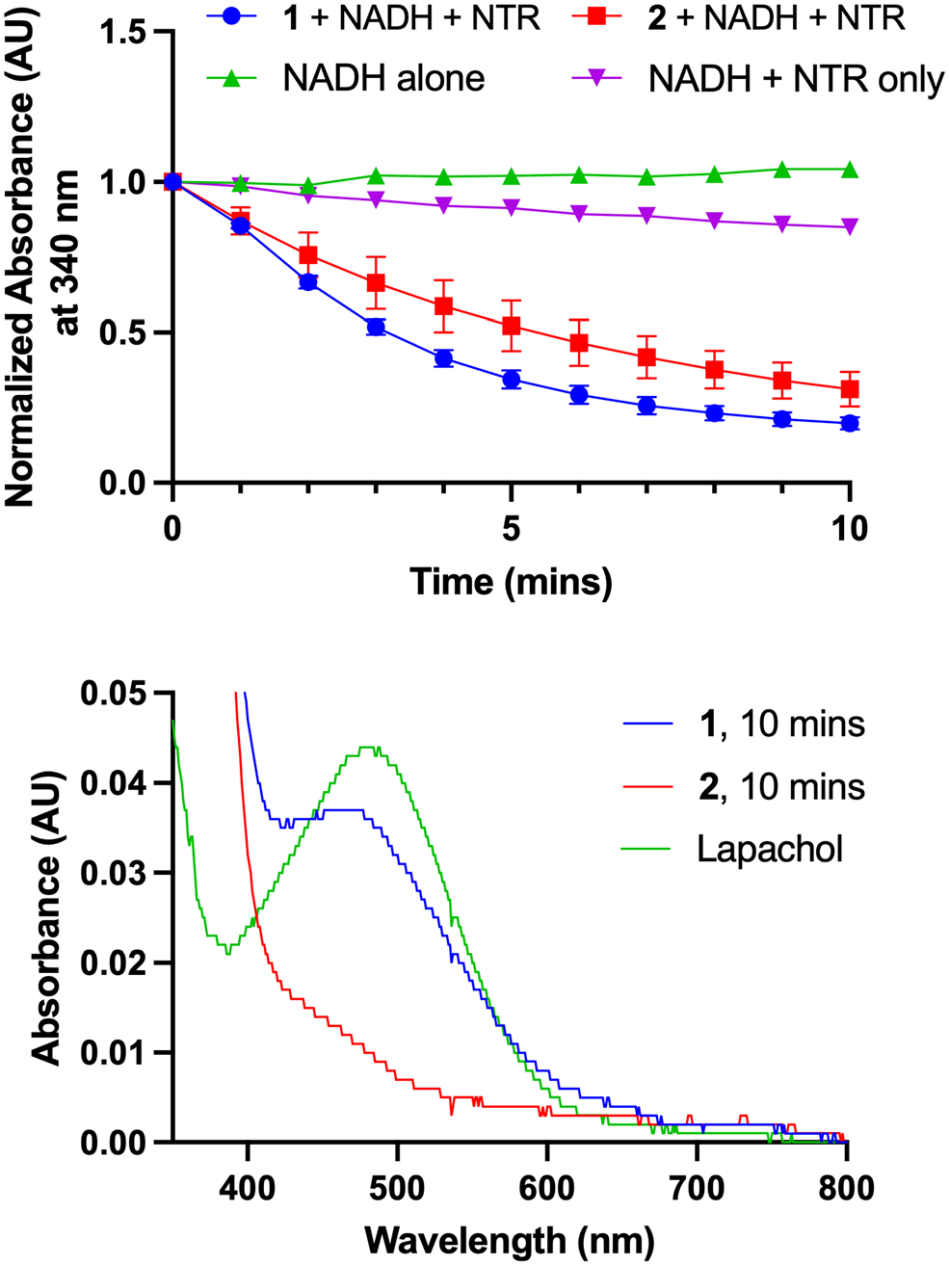
Top: Absorbance time course at 340 nm of 10 µM of **1** (blue) or **2** (red) in PBS (1% DMSO) with 500 nM NTR and 400 µM NADH. The control runs were 400 µM NADH alone (green) or NADH incubated with 500 nM NTR (purple) in PBS. Data presented as mean ± SD (*n* = 3). Bottom: Absorbance spectrum of **1** and **2** after incubated with NTR/NADH for 10 min. Only **1** acquired a new band at 480 nm (while **2** did not) which matches lapachol's characteristic peak (green).

To determine if reduction of the nitro group occurs readily upon incubation with NTR and NADH, the absorbance of NADH at 340 nm was monitored with time, since the resulting oxidized NAD^+^ does not absorb in this region. The NTR activities of **1** and **2** were evaluated, whereby a decrease in absorbance at 340 nm was observed with time for both **1** and **2**, indicative of NTR‐mediated reduction (Figure 5, Top).

We next determined whether lapachol was being released because of NTR‐mediated reduction of **1** and **2**. After ten minutes of coincubation of the compounds with NTR and NADH, the characteristic absorbance peak at 480 nm for lapachol emerged in **1** but was absent for **2** (Figure 5, Bottom). We further confirmed lapachol release from **1** after NTR incubation through HPLC and LC‐MS analysis (Figures S7–S9). Thus, as designed, we expect **1** to elicit antimicrobial activity, but not **2**.

**FIGURE 6:**
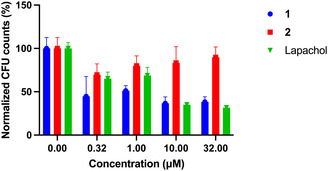
Normalized colony formation units of *B. subtilis* coincubated with increasing concentrations of **1**, **2**, or lapachol (2% DMSO). Bacteria were treated with increasing concentrations (0–32 µM) of respective compounds in the dark at 37°C, with treatment by **1** or lapachol resulting in a dose‐dependent response in viability. Data presented as mean ± SD (*n* = 3).

*B. subtilis* was then cultured and coincubated with increasing concentrations of **1**, **2**, or lapachol for 6 h and subsequently plated and colony formations counted. We observed **1** and lapachol having similar antimicrobial efficacy (50% colonies remaining at 10 µM), while **2** maintained a consistently high population even up to 32 µM (Figure 6). These findings indicate that **1** is indeed being converted to lapachol by NTR over a 6‐h period in *B. subtilis*, while **2**, though likely being reduced by NTR, is not releasing lapachol.

The minimum inhibitory concentration (MIC) of lapachol incubated with *B. subtilis* was 128 µM (or 31 µg/ml) (Figure S10), which is similar to a previously reported value of 64 µg/ml [15]. For **1** incubated with *B. subtilis*, we determined an MIC value of 1.024 mM (or 386 µg/ml) (Figure S10). For comparison, antibacterial agents such as Lincomycin and Retapamulin have reported MICs of 80 µg/ml [19]. Due to the initial lag in lapachol release from **1**, we hypothesize that a larger percentage of *B. subtilis* continues growing, resulting in the observed higher MIC value compared to lapachol and reported antibiotics.

**FIGURE 7:**
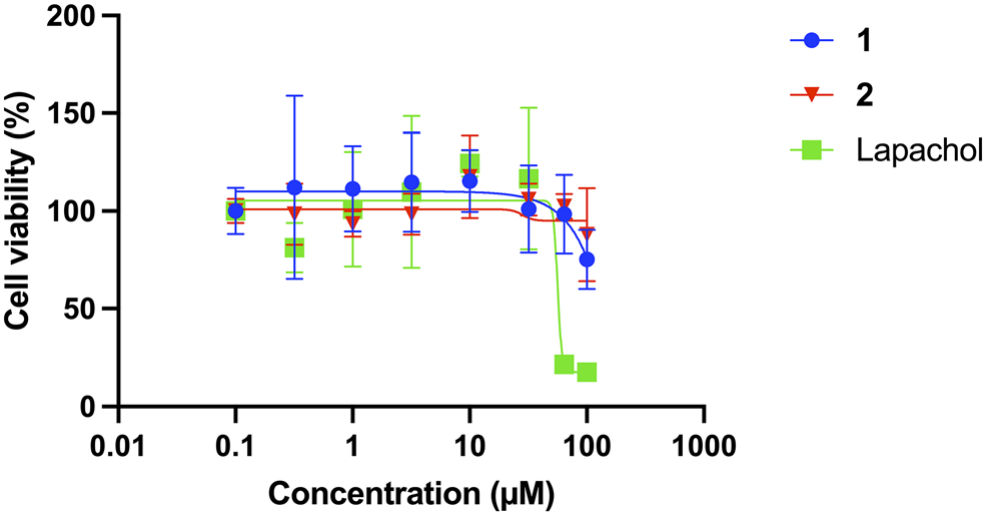
Cell viability of **1** and **2** compared to lapachol (2% DMSO) in MRC‐9 cells. Cells were treated with increasing concentrations (0–100 µM) of respective compounds in the dark at 37°C. Only treatment with lapachol resulted in a dose‐dependent response in viability. Data presented as mean ± SD (*n* = 3).

Once NTR‐triggered release of lapachol was confirmed in *B. subtilis*, compounds **1** and **2** were investigated and compared to lapachol in the lung fibroblast cell line, MRC‐9 (Figure 7), which will serve as a model healthy mammalian cell with no NTR expression. We found that treating MRC‐9 cells with increasing concentrations of **1** or **2** did not cause significant cell toxicity, even at 100 µM, while lapachol showed significant toxicity at 64 µM. Comparatively, since **1** in *B. subtilis* is most potent at 10 µM (Figure 6), it has a >10‐fold selectivity over mammalian cells, while free lapachol, though also most potent at 10 µM in *B. subtilis*, has poorer selectivity for bacteria over mammalian cells (6.4‐fold), suggesting that conversion of lapachol to the NTR prodrug **1**, does indeed improve antibacterial selectivity.

In summary, by utilizing lapachol as a natural product source for antimicrobial activity and appending the 4‐nitrobenzyl trigger group, a novel prodrug was designed and synthesized for the purpose of investigating new treatments for AMR. We accomplished NTR‐dependent activation by modifying lapachol's free hydroxyl with a nitrobenzyl moiety. Compound **1** showed high efficacy in *B. subtilis*, differentiating between bacterial and mammalian cells with high selectivity compared to free lapachol.

## Supporting Information

Additional supporting information can be found online in the Supporting Information section. **Supporting Fig. S1:** ^1^H NMR Spectrum for **1**. **Supporting Fig. S2:** ^13^C NMR Spectrum for **1**. **Supporting Fig. S3:** ^1^H NMR Spectrum for **2**. **Supporting Fig. S4:** ^13^C NMR Spectrum for **2**. **Supporting Fig. S5:** MS Spectrum of **1**. m/z calc for C_22_H_19_NO_5_^‐^ [M]^‐^ 376.13, found 376.13. **Supporting Fig. S6:** MS Spectrum of **2**. m/z calc for C_23_H_21_NO_5_^‐^ [M]^‐^ 390.14, found 390.11. **Supporting Fig. S7:** HPLC trace of **1** incubated with NTR (blue) compared to trace of lapachol alone. **Supporting Fig. S8:** MS of lapachol sample with an HPLC retention time of 28 minutes. m/z calc for C_15_H_14_O_3_^‐^ [M]^‐^ 241.09, found 241.01. **Supporting Fig. S9:** MS of compound **1** incubated with NTR sample with an HPLC retention time of 28 minutes. m/z calc for C_15_H_14_O_3_^‐^ [M]^‐^ 241.09, found 241.07. **Supporting Fig. S10:** MIC assay of B. subtilis incubated with varying concentrations (0–1.024 mM).

## Author Contributions

All authors have given approval to the final version of the manuscript. **Ivonne R. Lopez‐Miranda**, **Tianyi Ma**, **Joshua N. Milstein**, and **Andrew A. Beharry** designed experiments. **Ivonne R. Lopez‐Miranda** conducted synthesis and performed all experiments. **Tianyi Ma** performed the *B. subtilis* culture experiments. The manuscript was written by **Ivonne R. Lopez‐Miranda** and **Andrew A. Beharry** and edited by **Joshua N. Milstein**.

## Funding

This work was supported by NSERC Discovery Grants

## Conflicts of Interest

The authors declare no conflicts of interest.

## Supporting information

Supplementary Material

## Data Availability

The data that support the findings of this study are available from the corresponding author upon reasonable request.
