# Supplementary material for: A Nitroreductase‐Activatable Lapachol Against Bacillus subtilis Unveils Antimicrobial Specificity
Source: ChemMedChem. 2026 Mar 13;21(5):e202500997. doi: 10.1002/cmdc.202500997 (PMC12987600; doi:10.1002/cmdc.202500997)
Supplement: Supplementary file 1 — Supplementary Material [file CMDC-21-e202500997-s001.pdf]

A Nitroreductase-activatable Lapachol Against Bacillus Subtilis Unveils Antimicrobial Specificity

Supplemental Information

Ivonne R. Lopez-Miranda[1,2] Tianyi Ma[1,3], Joshua N. Milstein[1,3], and Andrew A. Beharry[1,2]*

[1]Department of Chemical and Physical Sciences, University of Toronto Mississauga, Mississauga, Ontario L5L 1C6, Canada.

[2]Department of Chemistry, University of Toronto, 80 St. George Street, Toronto, Ontario M5S 3H6, Canada

[3]Department of Physics, University of Toronto, 60 St. George Street, Toronto, Ontario M5S 1A7, Canada

Table of Contents

1. General Information ....

2. Experimental Procedures and Characterization Data.......................................

2.1. Experimental Procedures

2.2. Characterization Data ....

2.2.1. NMR Spectra ....

2.2.2. MS Spectra...

3. UV-Vis Characterization ....

4. *In vitro* NTR-mediated NADH reduction assay

5. NTR-activation assay monitored by HPLC

6. Bacterial strains and preparation

7. MIC assay, *B. subtilis*

8. Bacterial cell viability, *B. subtilis*

9. Mammalian Cell Culture...

10. Mammalian Cell Viability Assay ....

1. General Information

All reagents and solvents were purchased from commercial suppliers and used without further purification. Reactions were monitored by thin layer chromatography (TLC) using silica gel 60 F254 aluminum sheets and visualized under UV-C (254 nm) or UV-A (365 nm) light. Flash column chromatography was performed with Silicycle® Siliaflash® P60, 40-63 μM silica gel.

The ^{1}H and ^{13}C NMR spectra were recorded using a Bruker AV-III-400 (at an operating frequency of 400 MHz for ^{1}H and 100 MHz for ^{13}C NMR) at 298 K using deuterated solvents with tetramethylsilane (TMS) as internal standard. ^{1}H and ^{13}C Chemical shifts (δ) are reported in ppm relative to solvent residual signals ($CDCl_3$= 7.26 ppm for ^{1}H, 77.16 ppm for ^{13}C) (DMSO-*d*6 = 2.50 ppm for ^{1}H, 39.52 ppm for ^{13}C) (MeOD = 4.87 ppm for ^{1}H), and coupling constants reported in Hz. Data for ^{1}H NMR is reported as follows: chemical shift (δ ppm), multiplicity (s = singlet, d = doublet, t = triplet, q = quartet, m = multiplet, br = broad), coupling constant (Hz), integration.

Mass spectra were acquired using a Waters Quattro Ultima LC-MS with electrospray ionization (ESI).

2. Experimental Procedures and Characterization Data

2.1 Experimental Procedures

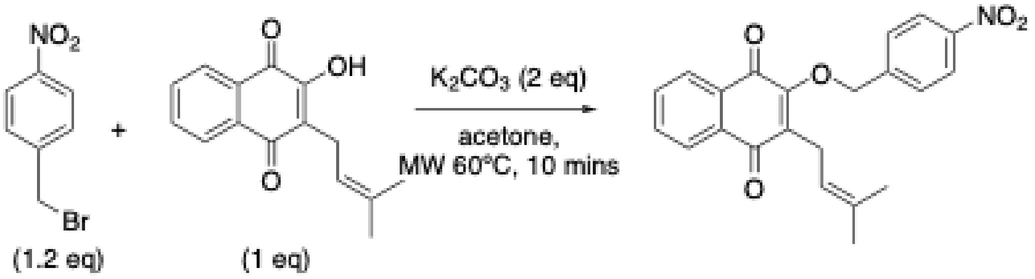

Synthesis of 2-(3-methylbut-2-en-1-yl)-3-((4-nitrobenzyl)oxy)naphthalene-1,4-dione (Compound **1**)

50 mg (0.206 mmol, 1 eq) of lapachol and 54 mg (0.248 mmol, 1.2 eq) of 4-nitrobenzyl bromide were added to a microwave-compatible glass vial. 57 mg (0.412 mmol, 2 eq) of potassium carbonate were added, and all were dissolved in acetone. The solution was microwaved at 60ºC for 10 minutes. The solution was extracted using DCM and water, and the organic fractions were washed with NH_4Cl. The organic fractions were dried in vacuo and purified using HPLC (5-100% ACN in 17.5 minutes in MQ water, using 0.1% formic acid, eluting at 24 minutes). 15 mg of yellow-orange solid was collected (20% yield). ^{1}H NMR (400 MHz, DMSO) δ 8.29 – 8.22 (m, 2H), 7.97 (ddd, *J* = 7.6, 5.6, 3.6 Hz, 2H), 7.78 (s, 2H), 7.74 (d, *J* = 8.4 Hz, 2H), 5.49 (s, 2H), 4.95 (t, *J* = 7.2 Hz, 1H), 3.15 (d, *J* = 7.2 Hz, 2H), 1.58 (s, 3H), 1.56 (s, 3H). ^{13}C NMR (101 MHz, DMSO) δ 184.55, 180.88, 156.38, 147.17, 144.70, 134.17, 133.77, 133.50, 132.65, 131.32, 131.13,

128.75, 125.90, 125.72, 123.56, 120.06, 73.11, 25.36, 22.65, 17.66. (ESI): m/z calc for $C_{22}H_{19}NO_5^-$ $[M]^-$ 376.13, found 376.13.

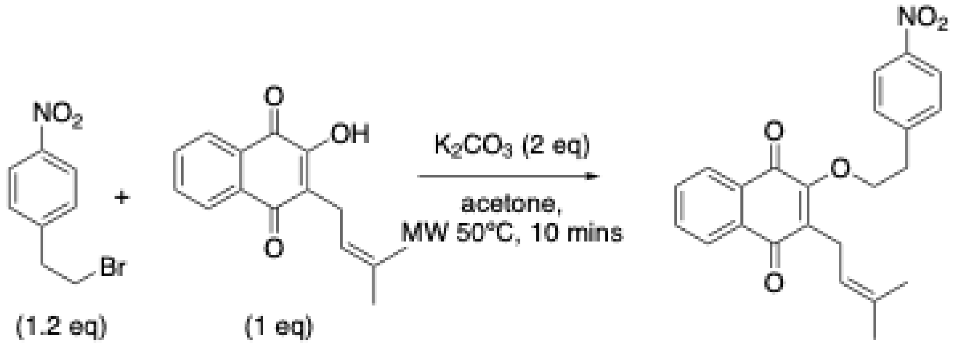

Synthesis of 2-(3-methylbut-2-en-1-yl)-3-(4-nitrophenethoxy)naphthalene-1,4-dione (Compound **2**)

10 mg (0.04 mmol, 1 eq) of lapachol and 12 mg (0.05, 1.2 eq) of 2-(4-nitrophenyl)ethylbromide were dissolved in acetone along with 11 mg (0.08, 2 eq) of potassium carbonate in a microwave-compatible glass vial and microwaved at 60ºC for 10 minutes. The solution was extracted using DCM and water, and the organic fractions were washed with NH_4Cl. The organic fractions were dried in vacuo and purified using HPLC (5-95% ACN in 17.5 minutes in MQ water, using 0.1% formic acid, eluting at 25 minutes). 2 mg of yellow solid was collected (8% yield). ^1H NMR (400 MHz, DMSO) δ 8.21 – 8.13 (m, 2H), 7.95 (dt, *J* = 4.9, 3.3 Hz, 2H), 7.87 – 7.77 (m, 2H), 7.63 (d, *J* = 8.6 Hz, 2H), 4.86 (t, *J* = 7.2 Hz, 1H), 4.68 (t, *J* = 6.3 Hz, 2H), 3.57 (s, 12H), 3.20 (t, *J* = 6.4 Hz, 2H), 3.03 (d, *J* = 7.1 Hz, 2H), 1.60 (s, 3H), 1.58 – 1.54 (m, 3H). ^{13}C NMR (101 MHz, $CDCl_3$) δ 145.83, 133.92, 133.81, 133.30, 129.93, 126.29, 126.11, 123.74, 119.82, 72.78, 36.83, 25.74, 23.04. (ESI): m/z calc for $C_{23}H_{21}NO_5^-$ $[M]^-$ 390.14, found 390.11.

2.2 Characterization Data

2.2.1 NMR Spectra

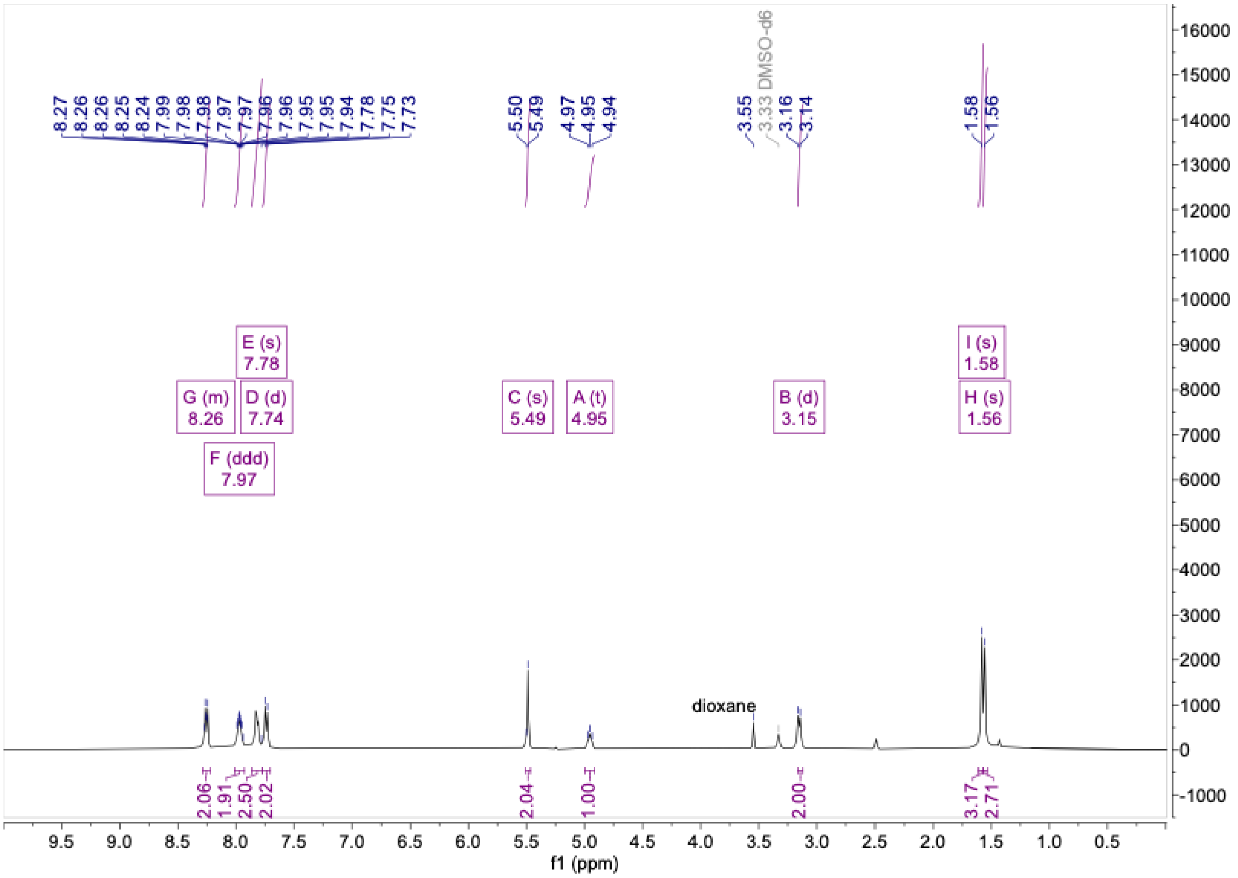

Figure S1. ^{1}H NMR Spectrum for **1**

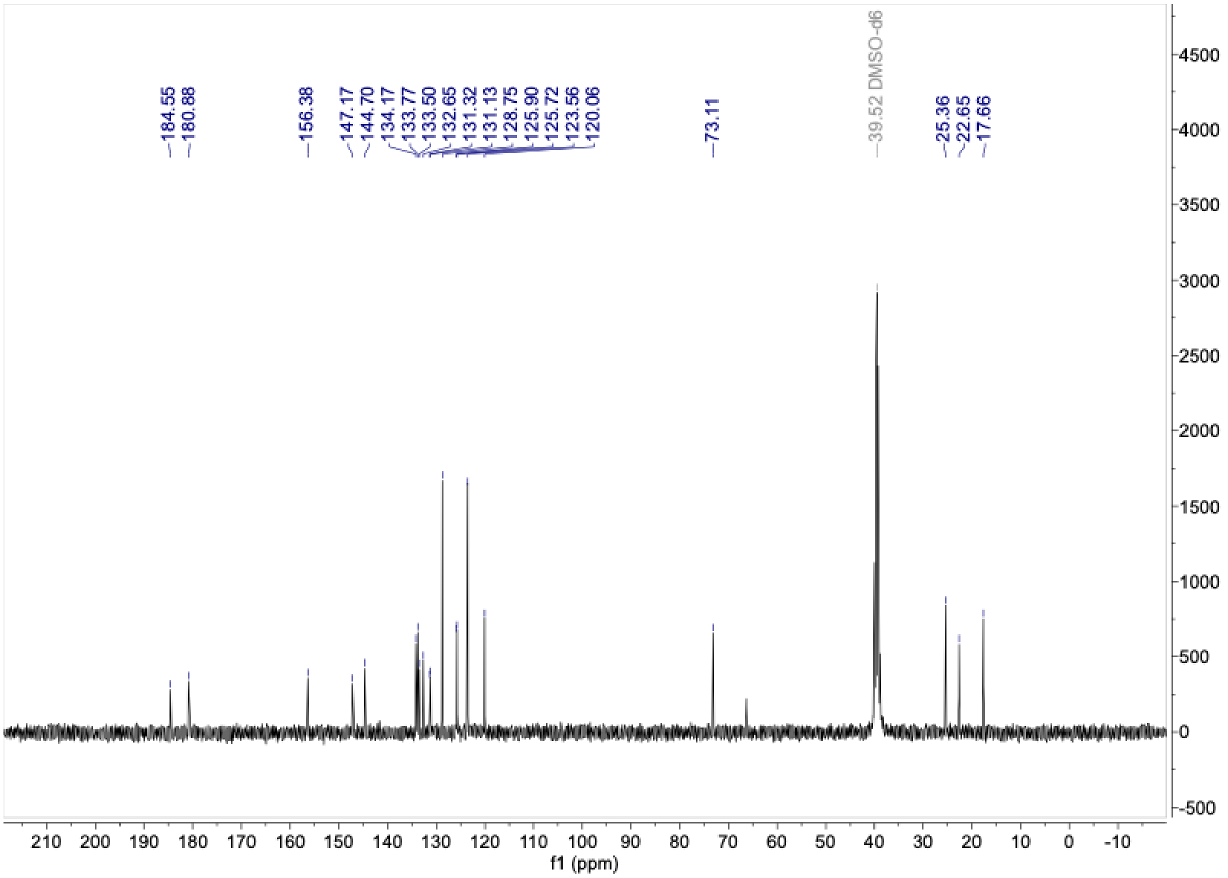

Figure S2. ^{13}C NMR Spectrum for **1**

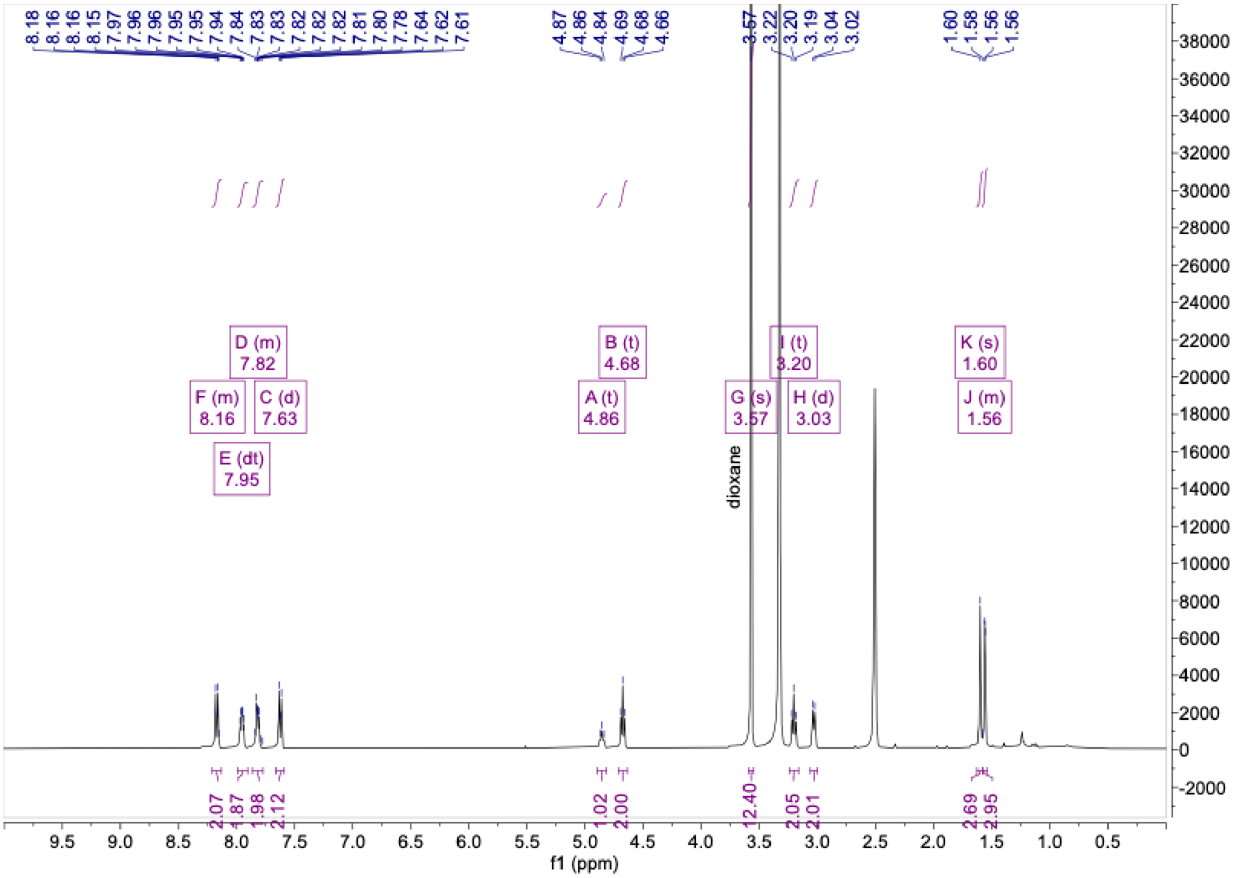

Figure S3. ¹H NMR Spectrum for **2**

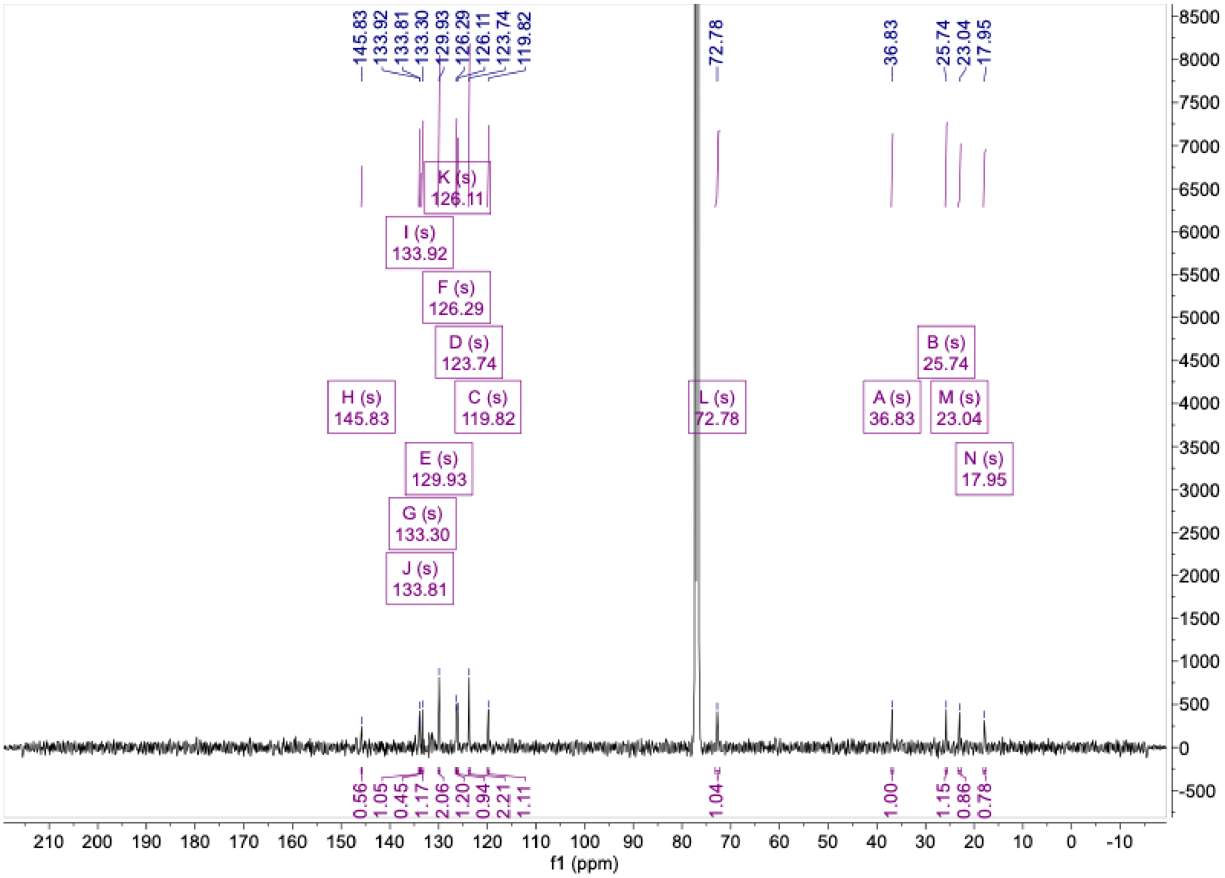

Figure S4. ^{13}C NMR Spectrum for **2**

2.2.2 MS Spectra

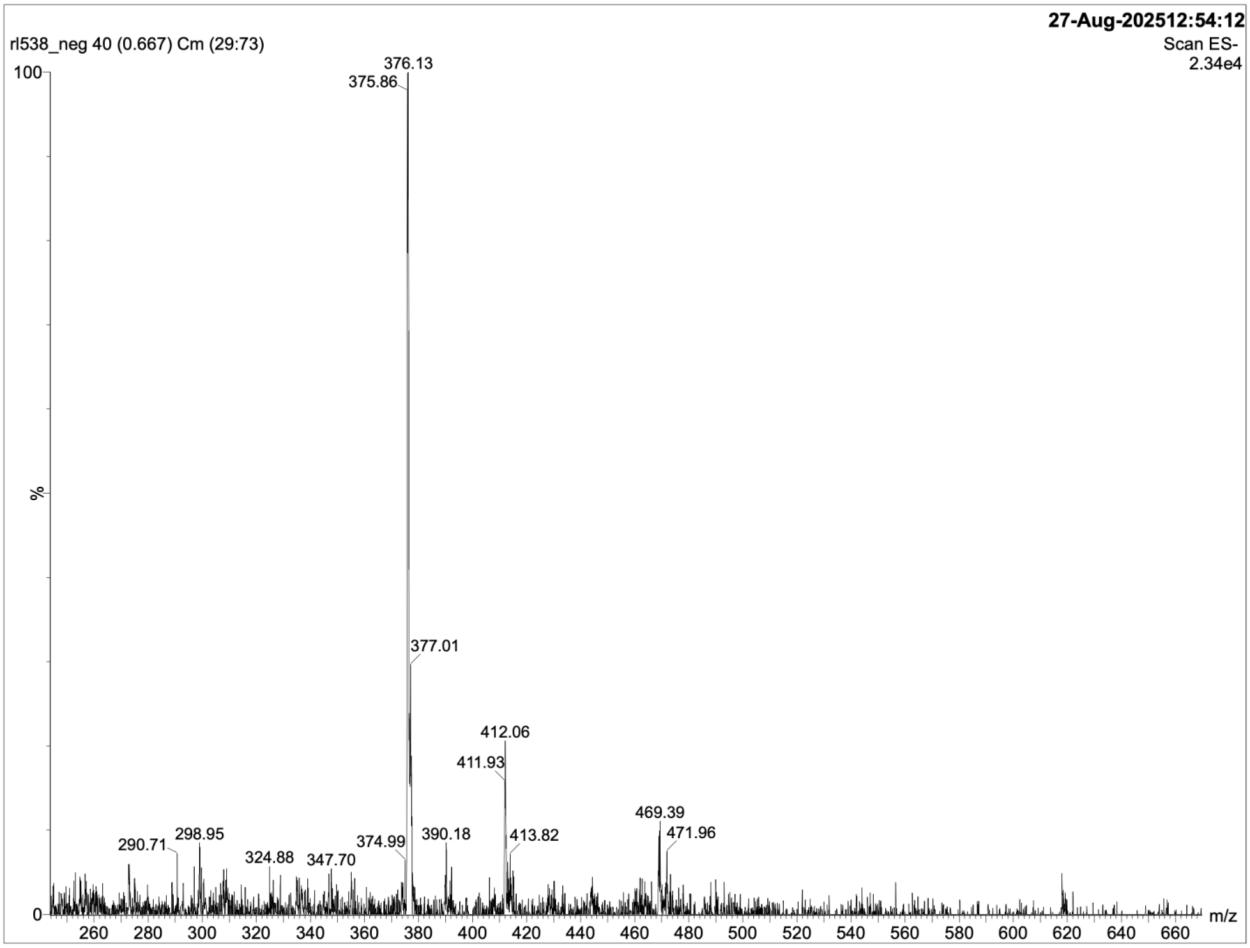

Figure S5. MS Spectrum of **1.** m/z calc for $C_{22}H_{19}NO_5^-$ $[M]^-$ 376.13, found 376.13.

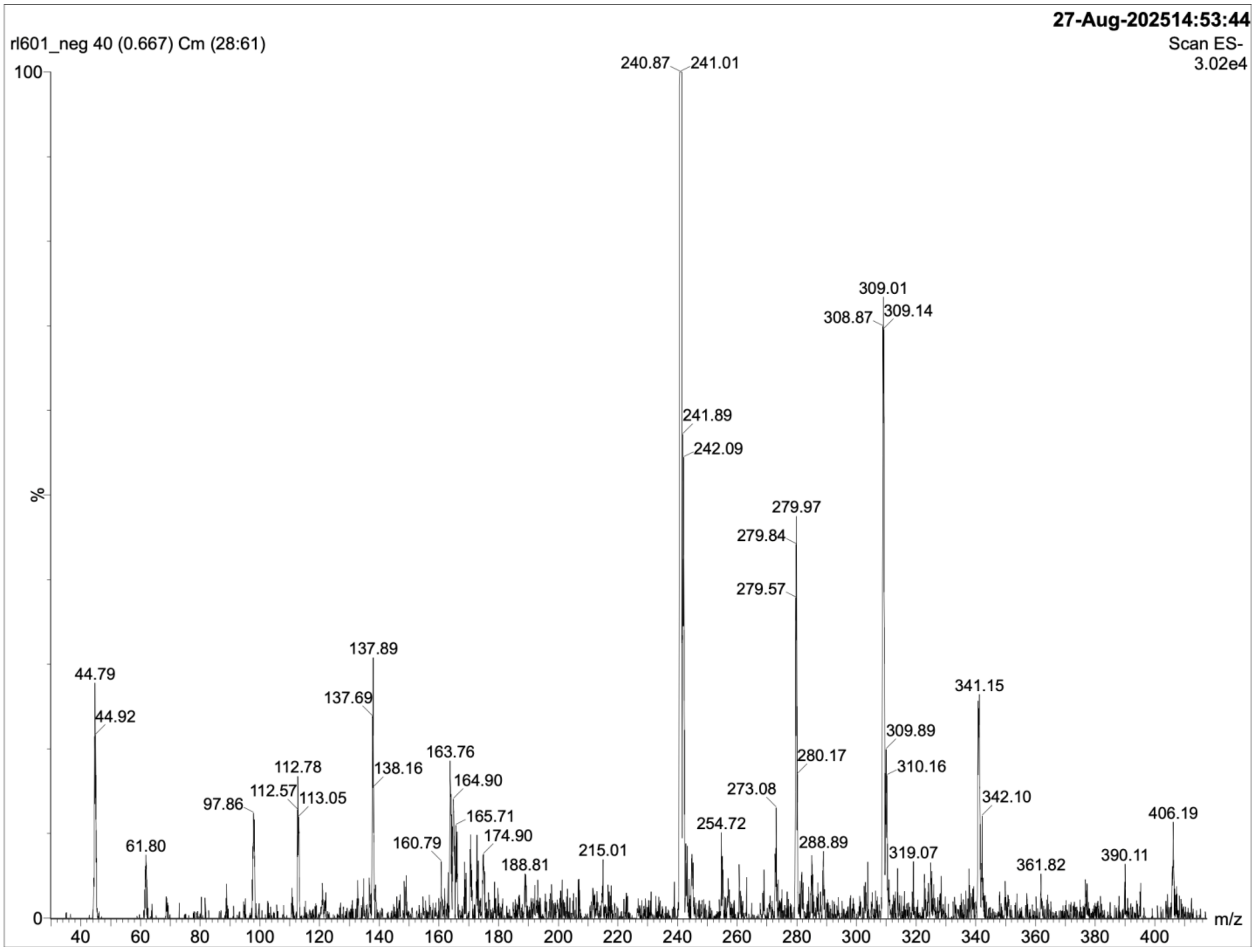

Figure S6. MS Spectrum of **2**. m/z calc for $C_{23}H_{21}NO_5^-$ $[M]^-$ 390.14, found 390.11.

3. UV-Vis Characterization

UV-VIS absorption spectra were recorded with a 1.0 cm path length cuvette on a Shimadzu UV-1800 UV-Visspectrophotometer.. Concentrated stock solutions of all compounds were made in DMSO and diluted 100x in the cuvette containing phosphate-buffered saline (PBS) for spectroscopic readings.

4. *In vitro* NTR-mediated NADH reduction assay

NTR substrates were evaluated using NADH recycling assay, in which NADH oxidation to NAD+ is monitored by absorbance at 340 nm on a Shimadzu UV-1800 Spectrophotometer. In a 60µL total volume cuvette, 10 µM of compounds 1 or 2 from 100x DMSO stock (0.6 µL) were added and diluted in 58.2µL of PBS (10mM, pH 7.4). NTR and NADH was added such that the final concentration of NTR (500 nM) (Sigma-Aldrich) and NADH (400 µM) (Sigma-Aldrich) was achieved. Absorbance change at 340 nm was measured at 1-minute intervals by scanning from 200-800 nm every minute at

37ºC. 3 replicates were measured each experiment. The control was NADH alone and NADH incubated with NTR only.

5. NTR-activation assay monitored by HPLC

To test for release of lapachol from compound **1** after NTR incubation, HPLC analysis of samples were performed. Compound **1** (1 mM, 2% DMSO) was incubated with NTR (500 nM) (Sigma-Aldrich) and NADH (400 µM) in PBS overnight at 37ºC. The sample was subjected to reverse-phase HPLC to identify products. HPLC was performed on a Shimadzu system run with an SPD-M20A VD diode array detector for detection on a Phenomenex Luna® C18 column (10 µm particle size, 100 Å pore size, 250 x 10 mm ID), equipped with a LC20AT Shimadzu pump and CBM20Z VP system controller. Solvent A: H2O with 0.1% FA, Solvent B: Acetonitrile with 0.1% FA. Gradient: 5% to 100% B in 12 minutes, 100% B for 9 minutes with 1.5 mL/min flow. Retention time for lapachol was 28 minutes. Peaks were collected and mass spectrometry analysis (Waters Quattro Ultima LC-MS , ESI$^-$) was conducted.

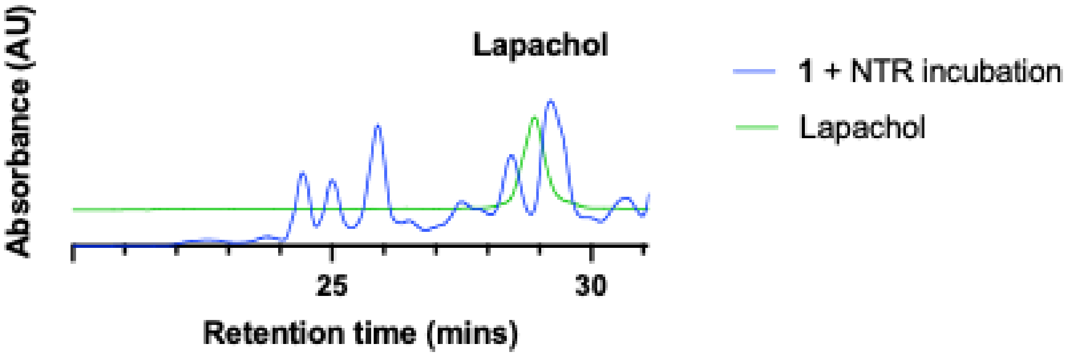

Figure S7. HPLC trace of **1** incubated with NTR (blue) compared to trace of lapachol alone.

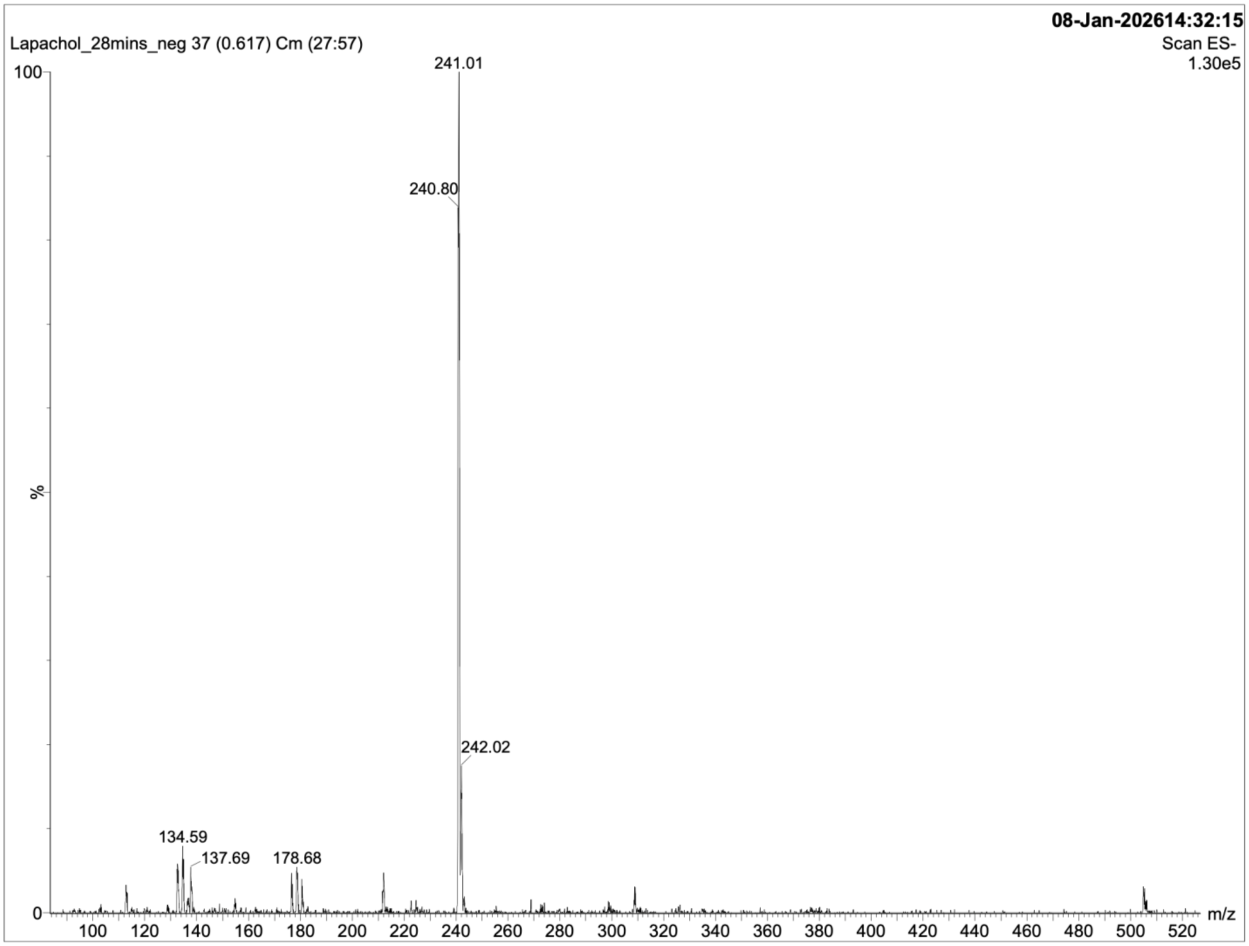

Figure S8. MS of lapachol sample with an HPLC retention time of 28 minutes.m/z calc for $C_{15}H_{14}O_3^-$ $[M]^-$ 241.09, found 241.01.

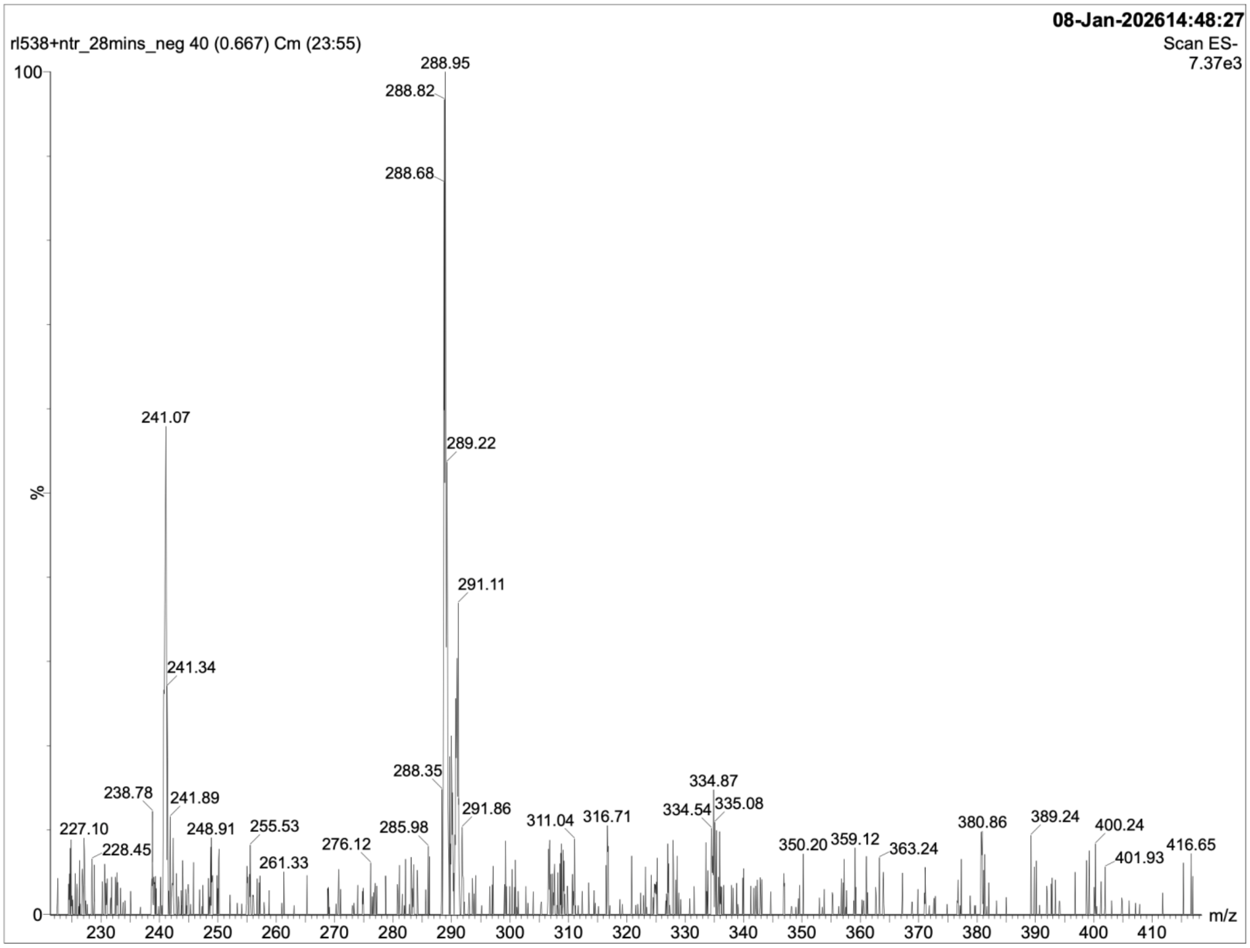

Figure S9. MS of compound **1** incubated with NTR sample with an HPLC retention time of 28 minutes.m/z calc for $C_{15}H_{14}O_3^-$ $[M]^-$ 241.09, found 241.07.

6. Bacterial strains and preparation

Colonies of *Bacillus Subtilis* 168 (wild-type) were thawed from frozen concentrated stocks and regrown in LB broth overnight to prepare the agar plate stocks for daily usage. On the day of experiments,*B. subtills* were inoculated from a colony into 5 mL of LB broth and grown at 37°C overnight with shaking at 250 rpm. The next day, bacteria were regrown by diluting the overnight culture 100× into fresh LB broth (10 mL total volume). Bacteria were grown at 37°C for an additional 2 hours with shaking at 250 rpm until they reached an exponential phase (OD_{600}= 0.10–0.50). Standardized suspensions of bacteria were prepared for all experiments to the same OD_{600}.

7. MIC assay, *B. subtilis*

After culturing and regrowing *B. subtilis*, an initial OD_{600}= 0.1 AU was used and incubated with **1** and lapachol (2.048 mM, final 2% DMSO) and incubated at 37ºC with shaking at 200 rpm for 7 hours. The OD_{600} of the untreated stock was measured, and the sample was diluted to have an OD_{600} = 0.1, which was diluted 100x into fresh LB broth (10 mL total volume). The 2.048 mM stocks of **1** and lapachol were diluted 2x with

fresh LB broth in each subsequent row of a 96-well plate, resulting in varying concentrations (0–1.024 mM) of compound used to treat *B. subtilis* which was then added from the stock solution into the wells. A growth control (no compound added to *B. subtilis*) and sterile control (LB broth alone with no *B. subtilis*) were also prepared. The plate was incubated overnight at 37ºC with shaking at 200 rpm. Absorbance values at 600 nm of each well were read by a Tecan Infinite M1000 plate reader. Cell viability of wells was performed in triplicate. Data was analyzed with GraphPad Prism 10.

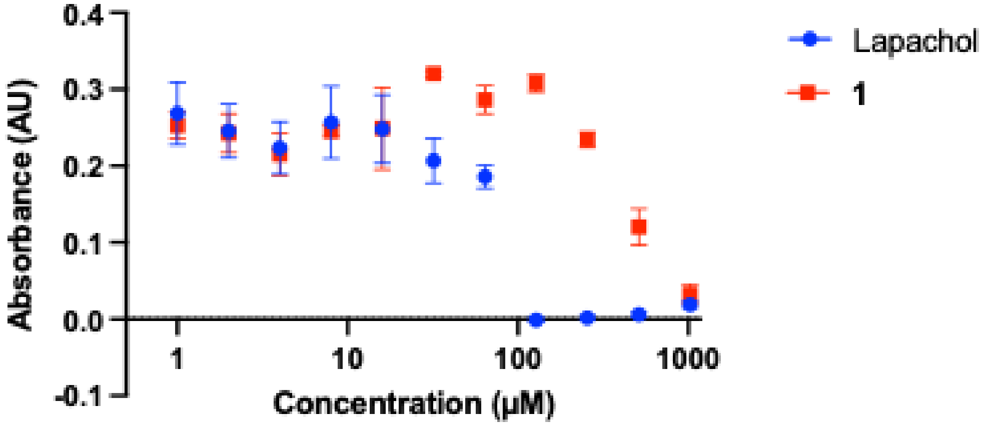

Figure S10. MIC assay of *B. subtilis* incubated with varying concentrations (0–1.024 mM)

8. Bacterial cell viability,*B. subtilis*

After culturing and regrowing *B. subtilis*, an initial OD_{600}=0.05 AU was used and incubated withvarying concentrations (0–32 μM) of **1**, **2**, and lapachol (final 2% DMSO) and incubated in the dark at room temperature for 6 hours. The $OD_{600}$of the dark control (*B. subtilis*with 2% DMSO alone) was measured to calculate the dilution factor required to reach a final OD_{600} of $1.5\times10^{-4}$for plating. All samples were diluted with LB broth using the same dilution factor and 5 μL of the diluted sample was spread using glass beads onto 6-well cell culture plates (35 mm diameter), prepared previously by melting LB agar (2 mL each well) and leaving to solidify at room temperature. Plates were left to incubate at 37 °C overnight before counting the colonies formed. Experiments were conducted in triplicate.Colony formed plates were imaged with iBrightFL1500. Cell Counter plug-in from FIJI was used to manually count the individual colonies formed on the agar plates.

CFU data:

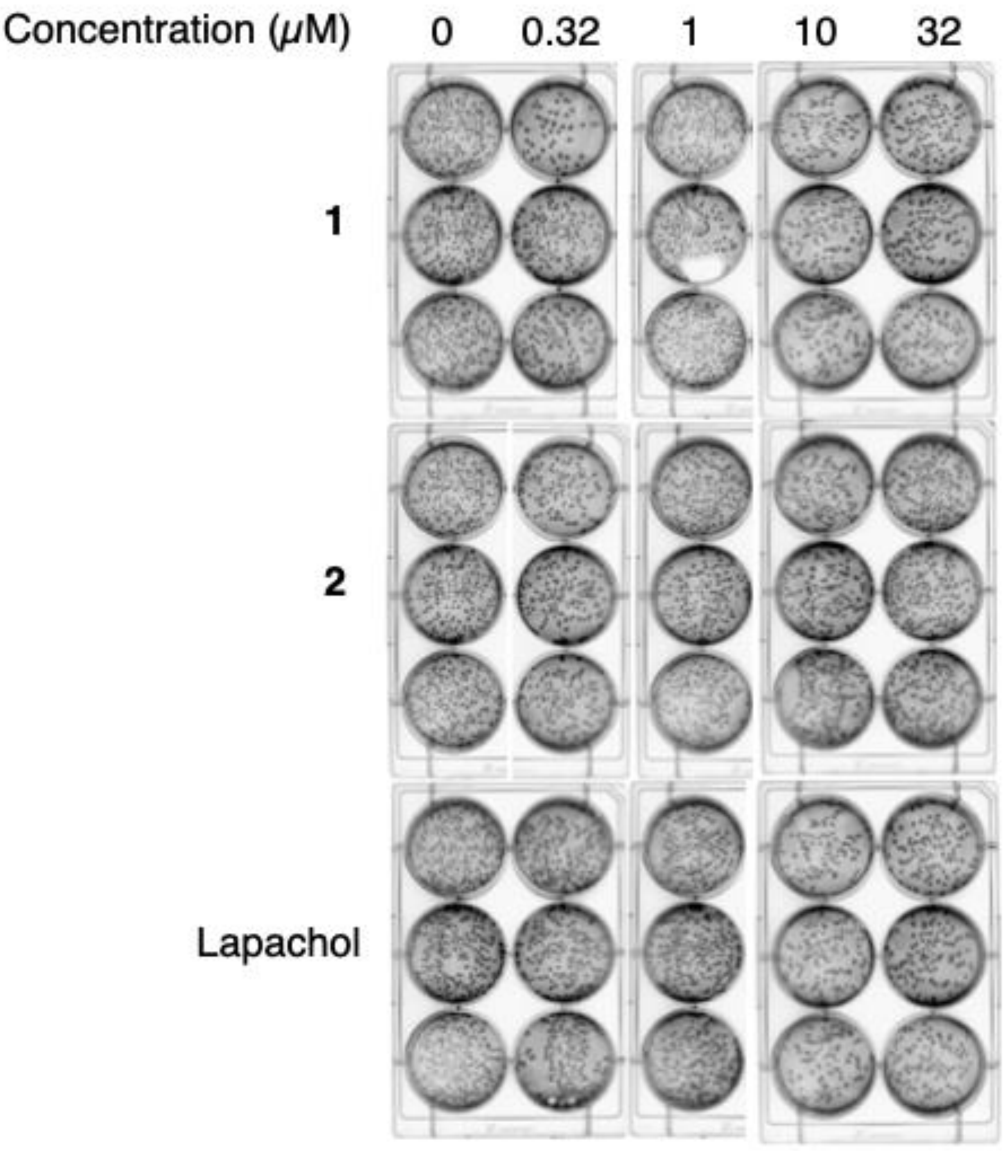

1				Average	STD	Normalized Average	Normalized STD
0	258	222	222	234	20.7846097	100	12.5614859
0.32	48	152	114	104.666667	52.6244557	44.7293447	22.8373293
1	159	132	159	150	15.5884573	51.1945392	5.86351231
10	115	124	84	107.666667	20.984121	36.7463026	7.37710069
32	94	124	119	112.333333	16.0727513	38.3390216	5.78781588

2				Average	STD	Normalized Average	Normalized STD
0	258	222	222	234	20.7846097	100	12.5614859
0.32	133	176	179	162.666667	25.7358375	69.5156695	12.6129511
1	236	202	265	234.333333	31.5330514	79.9772469	11.430242
10	218	208	306	244	53.9258751	83.2764505	18.8363931
32	283	281	226	263.333333	32.3470761	89.8748578	11.8576759

Lapachol				Average	STD	Normalized Average	Normalized STD
0	306	278	295	293	14.106736	100	6.80885234
0.32	200	205	168	191	20.0748599	65.1877133	7.53612534
1	202	227	176	201.666667	25.5016339	68.8282139	9.31312996
10	102	107	99	102.666667	4.04145188	35.039818	2.17913028
32	96	90	93	93	3	31.7406143	1.83947916

9. Mammalian Cell Culture

MRC9 (ATCC) cells were cultured with Dulbecco's Modified Eagle's medium (DMEM) (Sigma-Aldrich, D6429). 10% FBS and 1% antibiotic-anti-mitotic (A.A.) solution was used. Cells were grown in T75 flasks and were maintained in a 5% CO_2 atmosphere at 37ºC.

10. Mammalian Cell Viability Assay

Cells were seeded at a density of 10,000 cells per well in 96-well plates (Thermo Scientific Nunclon™Delta Surface) and incubated with 200 µL appropriate complete growth medium overnight at 37 °C in 5% CO2 atmosphere. Varying concentrations (0–100 µM) of 1, 2, and lapachol were added (final 2% DMSO). The plates were then incubated overnight, after which 20 µL of 5 mg/mL solution of 3-(4,5-dimethylthiazol-2-yl)-2,5- diphneyltetrazolium bromide (MTT) (Alfa Aesar) in PBS was added to each well. Following 3 hours of incubation, the solution was removed and replaced with 100 µL of DMSO to suspend the formazan residue. Absorbance values at 565 nm of each well was read by a Tecan Infinite M1000 plate reader. Cell viability was calculated by; CV = $(A_{sample}/A_{control})$*100%, where A is the absorbance values of the wells treated with compound or the control group at 560 nm, with cell viability of the control group normalized to 100%. Cell viability of wells was performed in triplicate. Data was analyzed with GraphPad Prism 10.
